# Supplementary material for: Absence of warmth permits epigenetic memory of winter in Arabidopsis
Source: Nat Commun. 2018 Feb 12;9:639. doi: 10.1038/s41467-018-03065-7 (PMC5809604; doi:10.1038/s41467-018-03065-7)
Supplement: Supplementary file 1 — Supplementary Information [file 41467_2018_3065_MOESM1_ESM.pdf]

Supplementary Information for:

Absence of warmth permits epigenetic memory of winter in Arabidopsis

Hepworth, Antoniou-Kourounioti *et al.*

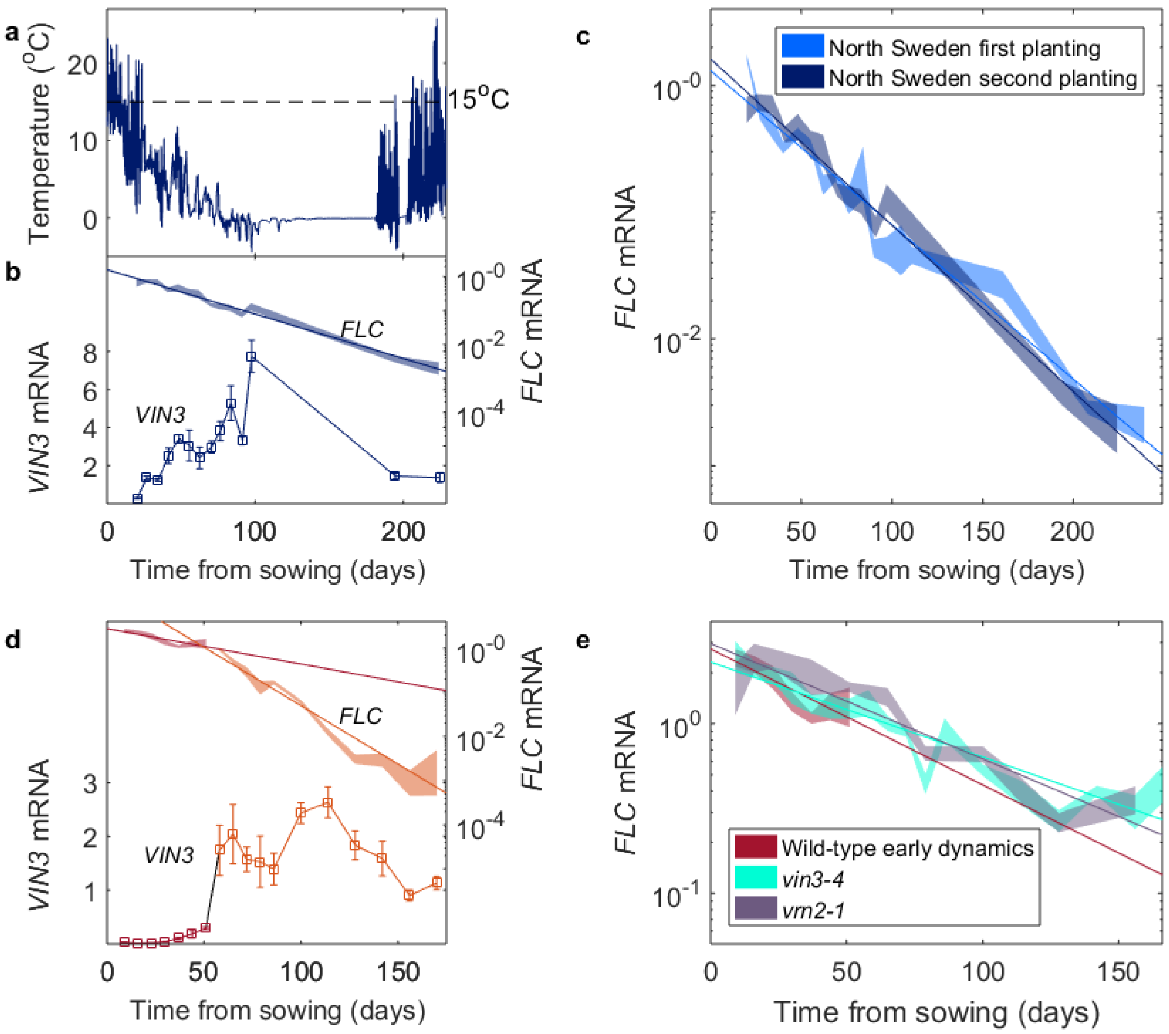

Supplementary Figure 1: Second planting in North Sweden behaves similarly to first and VIN3 separates the *FLC* shutdown in Norwich into two phases; the first phase being PRC2 independent.

a, Temperature for North Sweden second planting. The plants were sown 2 weeks later but otherwise experienced the same conditions as for the first planting. Dashed line indicates 15°C. **b**, *FLC* and *VIN3* expression for laboratory accession Col FRI^{SF2} as measured in the North Sweden second planting. *FLC* (top): Thick lines show *FLC* mRNA where (**b-e**) the thickness of the line shows the s.e.m. The straight line shows the best fit exponential decay curve, fitted by least-squares. *VIN3* (bottom): measured expression of *VIN3* mRNA; errors= s.e.m. **c**, Comparison of *FLC* downregulation between first and second planting in the North Sweden site. **d**, *FLC* and *VIN3* expression in Norwich separated according to presence (late) or absence (early) of *VIN3*; errors= s.e.m. **e**, *FLC* expression in Norwich in the wild-type (Col FRI^{SF2}) before *VIN3* upregulation and for the full duration of the experiment in the mutants *vin3-4* and *vrn2-1* which are deficient in *VIN3* expression and PRC2-mediated silencing respectively. RNA levels in **b-e** normalised to *UBC*, *PP2A*, and internal control. Straight lines (**b-e**) show the best fit exponential decay profile, fitted by least-squares. n=3-6, average ≥5.

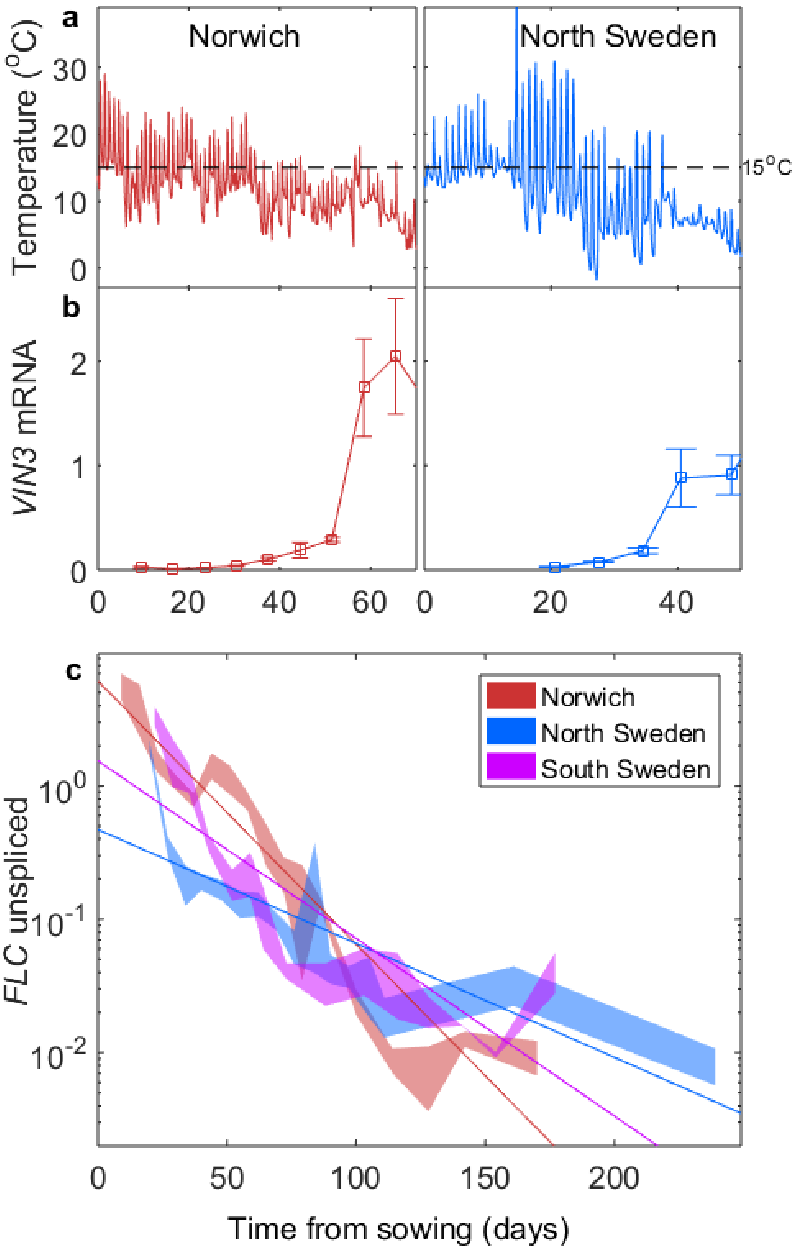

Supplementary Figure 2: Absence of warmth controls *VIN3* levels in the field.

a, Temperature in Norwich and North Sweden (first planting) in the early stages of the field experiment. Dashed line indicates 15°C. **b,** *VIN3* expression for Col *FRI*^{SF2} in the field sites corresponding to the plot above (data from Fig. 1); errors= s.e.m. **c,** Unspliced *FLC* levels from plants in the different field sites. Thick lines show measured expression levels normalised to *UBC*, *PP2A*, and internal control, where the thickness of the line represents s.e.m. Straight lines show the best fit exponential decay profile, fitted by least-squares. n=2-6, average ≥4.8 (data with single replicate are not shown).

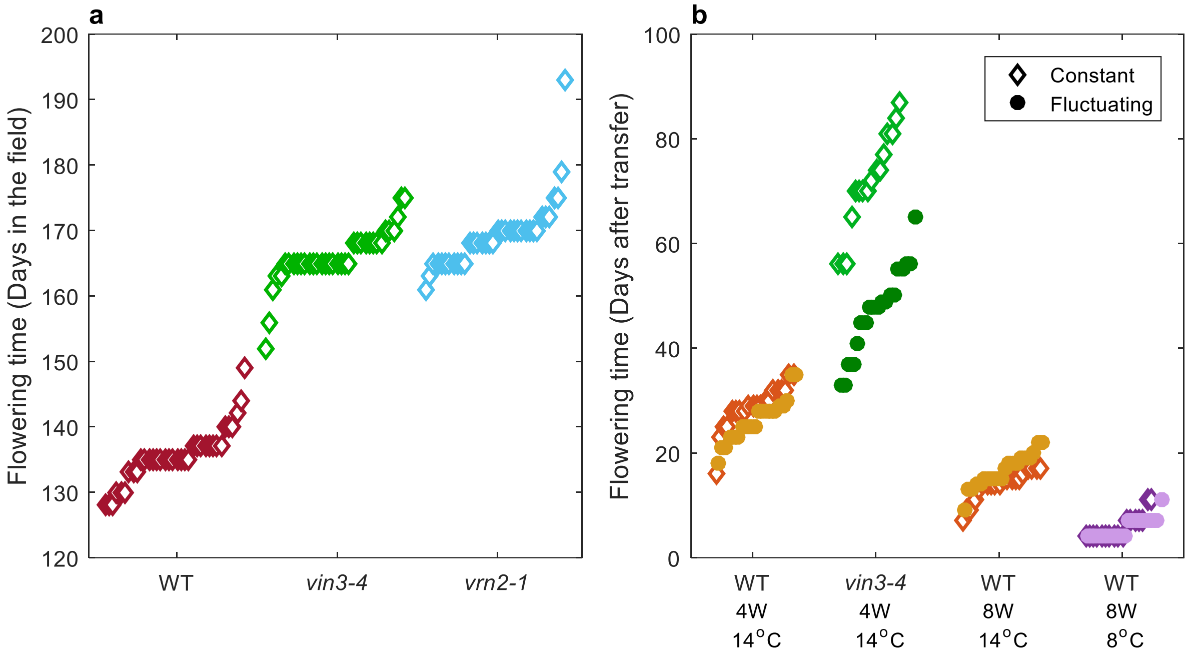

Supplementary Figure 3: Both the VIN3-dependent and independent pathways promote flowering, and absence of the VIN3-dependent pathway delays flowering in the field.

a, Flowering time after sowing in the field in Norwich 2014-15. WT = Col *FRI* genotype. n=36. **b,** Flowering time after transfer to 22°C, long day, floral inductive conditions for plants transferred after 4 or 8 weeks (W) vernalization in the temperature conditions shown in Fig. 2a. n=16-20, average≥18.8.

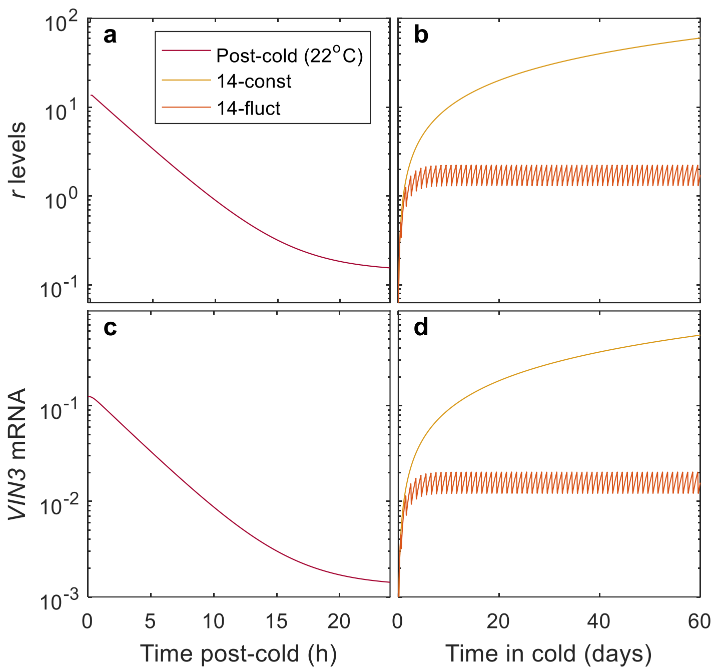

Supplementary Figure 4: *VIN3* dynamics follow regulator r

a-b, The predicted levels of regulator r for the model of Fig. 3a under the conditions described in Fig. 3c-d and Fig. 3b, respectively. The units of r are arbitrary. **c-d,** The predicted levels of *VIN3* under the same conditions.

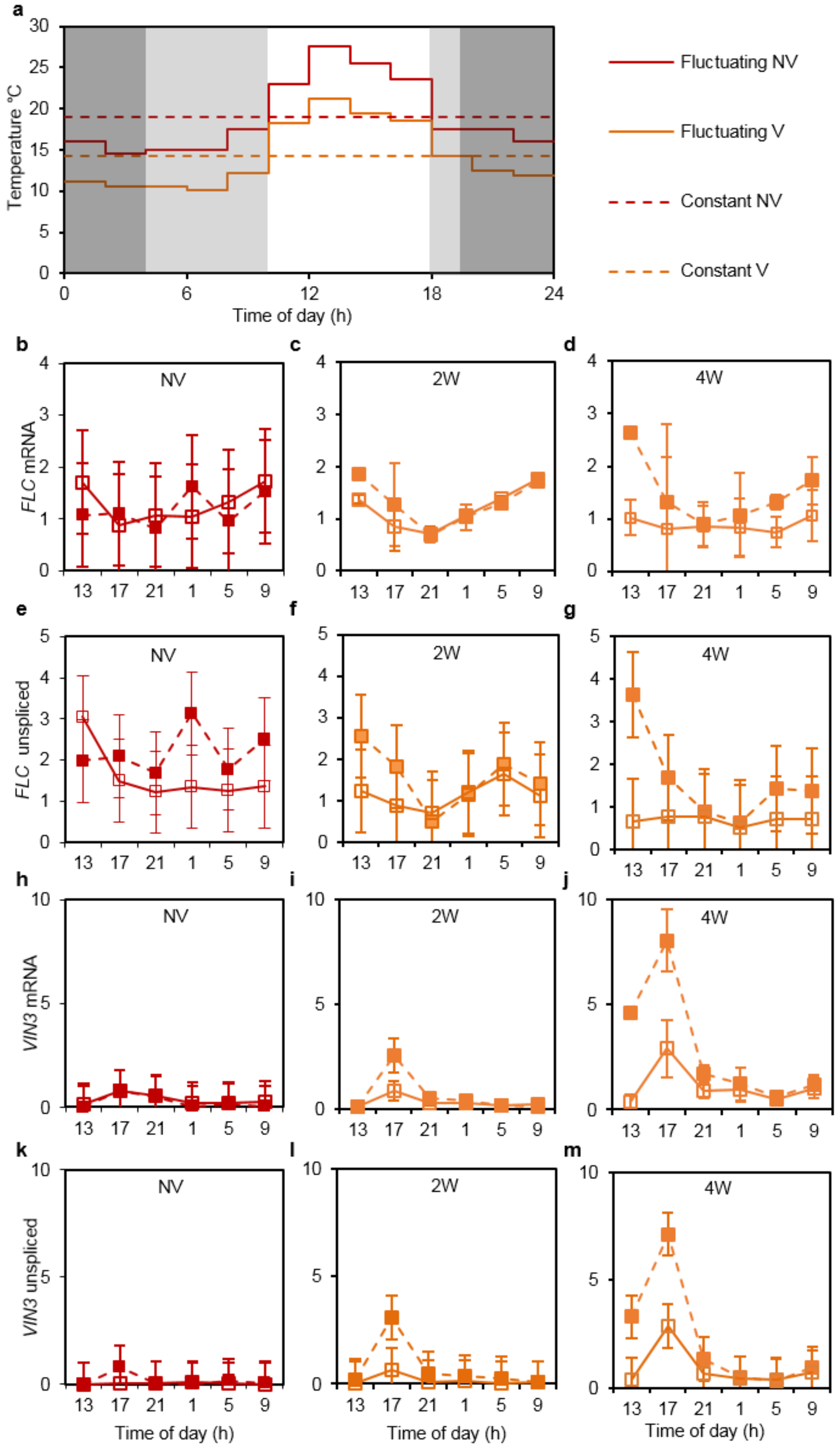

Supplementary Figure 5: *VIN3* expression shows a diurnal pattern over 24 hours, with a peak of expression that increases over weeks in the cold, whereas *FLC* expression does not show a diurnal pattern and is slowly repressed by cold.

a, Temperature profiles for plants grown in 16h photoperiod 'non-vernalizing' (NV) average 19°C conditions (constant or fluctuating) for one week, then transferred to 8h photoperiod 'vernalizing' (V) average 14.2°C conditions (constant or fluctuating, respectively). Dark shading for dark hours in NV, lighter shading for additional dark hours in V. **b-m**, Gene expression over 24 hours for plants grown in NV conditions of panel **a** for one week (constant or fluctuating) then transferred to V (constant or fluctuating, respectively) for time indicated. n=1-3, average >2.3. **b-d,** spliced *FLC* expression, **e-g**, unspliced *FLC* expression, **h-j,** spliced *VIN3* expression, **k-m**, unspliced *VIN3* expression. RNA levels are relative to *UBC*, *PP2A* and a control sample. 2W, 4W, 8W, indicates number of weeks in 'V' conditions at day of sampling. Error bars are s.e.m.

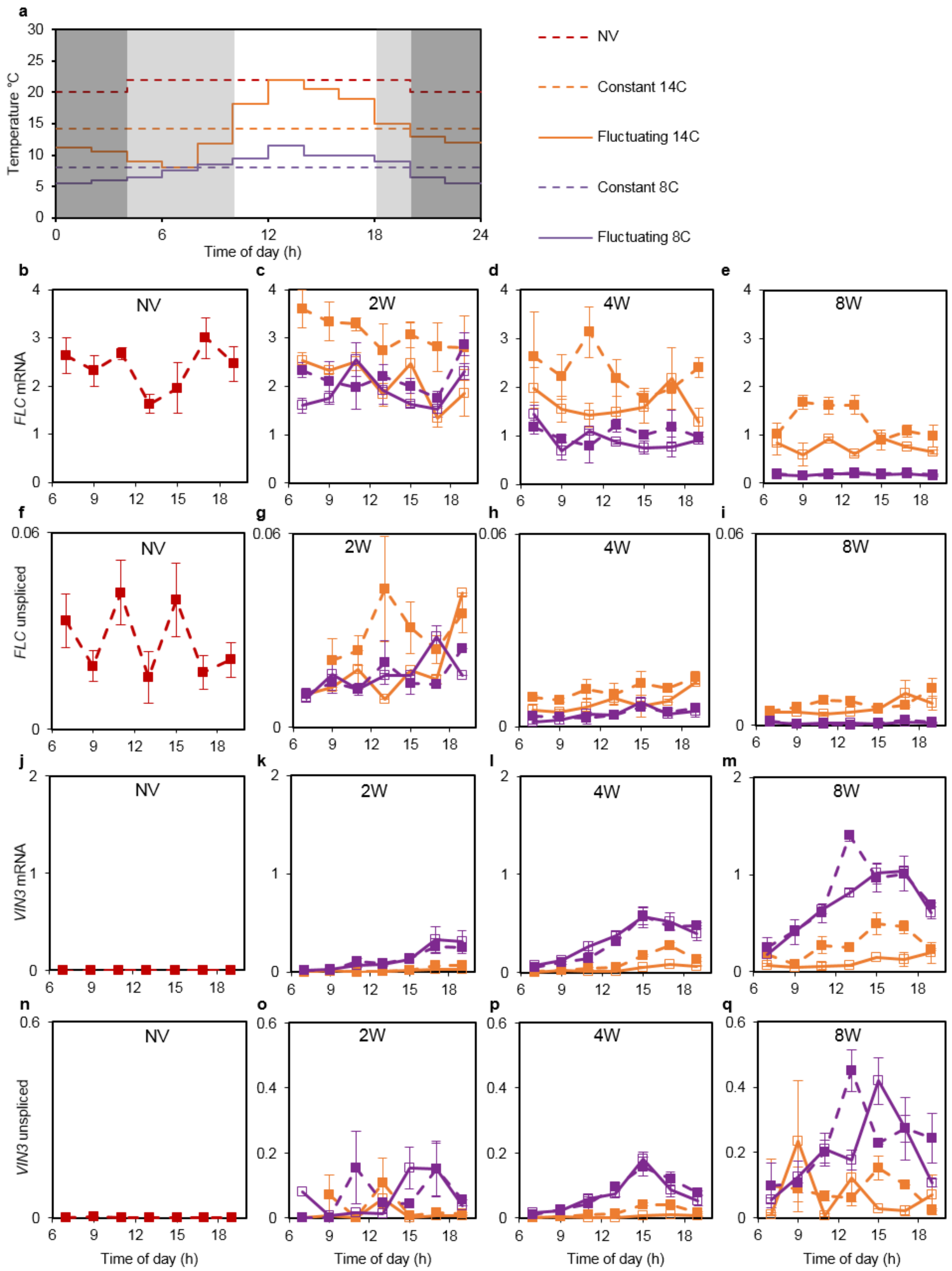

Supplementary Figure 6: *VIN3* expression shows a diurnal pattern, with a peak of expression that increases over weeks in the cold, whereas *FLC* expression does not show a diurnal pattern and is slowly repressed by cold.

a, Temperature profiles for plants grown in 20°C night, 22°C day long photoperiod for one week (NV) and transferred to 'V' short photoperiod conditions at average 8°C or 14.2°C profiles (constant or fluctuating), for time indicated. Dark shading for dark hours in NV, lighter shading for additional dark hours in V. **b-q,** Gene expression sampled during the day for plants grown as in panel **a**. n=1-3, average >2.7. **b-e**, spliced *FLC* expression, **f-i**, unspliced *FLC* expression, **j-m**, spliced *VIN3* expression, **n-q,** unspliced *VIN3* expression. RNA levels are relative to *UBC*, *PP2A*. 2W, 4W, 8W, indicates number of weeks in 'V' conditions at day of sampling. Error bars are s.e.m.

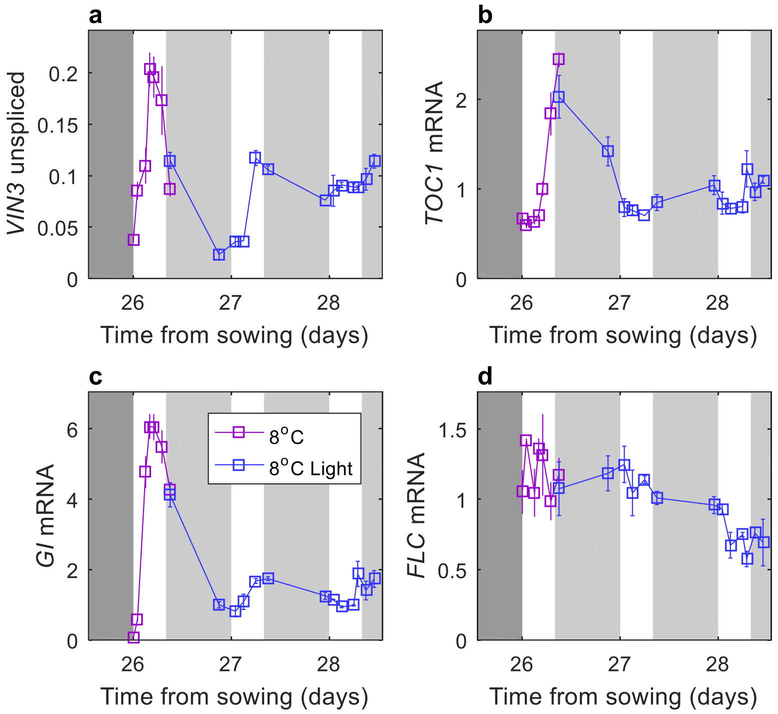

Supplementary Figure 7: Unspliced *VIN3* expression and mRNA levels for *TOC1*, *GI* and *FLC* over three days at the transition between short days to constant light.

a-d, RNA measurements (normalised to *UBC*, *PP2A*) from plants sampled over consecutive days at constant 8°C as in Fig. 4c-f (purple). On the first day of sampling the plants were transferred to constant light conditions (blue). The dark grey background indicates night-time, the lighter grey background indicates subjective night. Error bars are s.e.m. n=2-3, average >2.1.

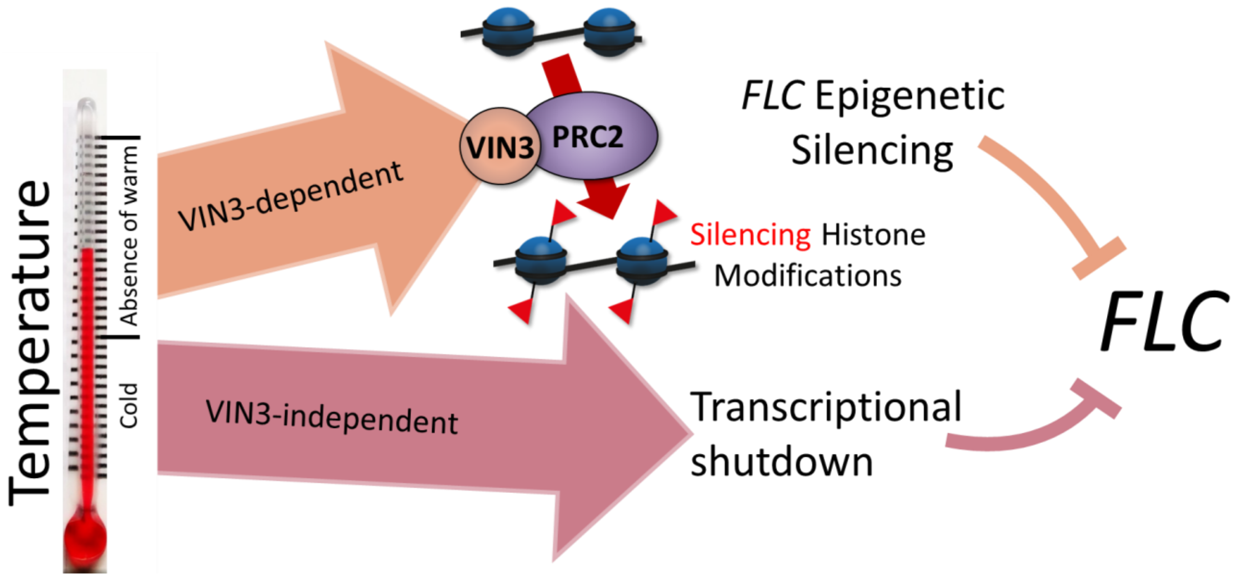

Supplementary Figure 8: Summary diagram of *FLC* temperature sensing.

Two pathways control *FLC* transcription in response to temperature. Transient cool temperatures activate the non-epigenetic “VIN3-independent” transcriptional silencing. The epigenetic pathway is triggered by the absence of warm temperatures which induces *VIN3* and allows the digital, cell-autonomous switching of *FLC* to the silenced state. The combination of these signals is interpreted by the plant as “winter”.

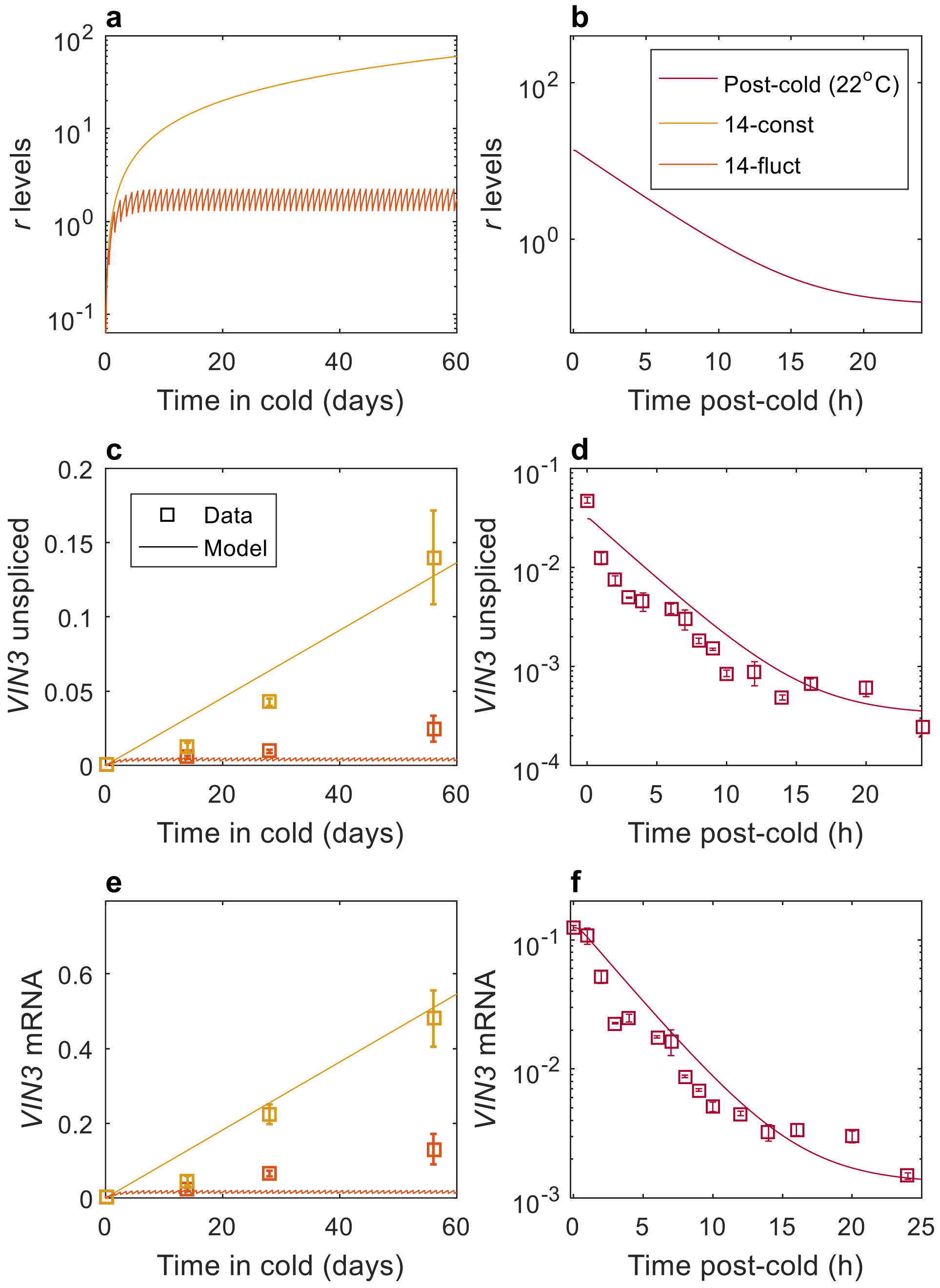

Supplementary Figure 9: Mathematical model with one thermosensor that explicitly includes unspliced *VIN3* also cannot capture the observed behaviour in fluctuating conditions.

a-f, Model predictions compared to data for the model that includes unspliced *VIN3* as an intermediate state. The equations for $\boldsymbol{r}$ and $d_r(T)$ are as shown in Fig. 3a**.** For unspliced *VIN3* ($\boldsymbol{u}$) the rate of change is given by $\frac{du}{dt} = p_1 r - s_1 u$ and for spliced *VIN3* ($\boldsymbol{v}$) it is $\frac{dv}{dt} = s_1 u - d_1 v$. Parameter values are $p_1 = 0.91\ \text{day}^{-1}, s_1 = 400\ \text{day}^{-1}, d_1 = 100\ \text{day}^{-1}, d_2 = 7\ \text{day}^{-1}$, initial conditions are $v_0 = 0, u_0 = 0, r_0 = 0$ for **a**, **c**, **e** and $v_0 = 0.124, u_0 = \frac{d_1 v_0}{s_1}, r_0 = \frac{d_1 v_0}{p_1}$ for **b**, **d**, **f**. **a-b**, The predicted levels of regulator $\boldsymbol{r}$ under the conditions described in Fig. 3b and Fig. 3c-d respectively. The units of $\boldsymbol{r}$ are arbitrary. **c-f,** The predicted levels of unspliced *VIN3* (**c-d**) and spliced *VIN3* (**e-f**) under the same conditions, compared to the data shown in Fig. 3b-c and Fig. 3d-e respectively. RNA levels were normalised to *UBC*, *PP2A*; error bars = s.e.m.
