# Supplementary material for: Absence of warmth permits epigenetic memory of winter in Arabidopsis
Source: Nat Commun. 2018 Feb 12;9:639. doi: 10.1038/s41467-018-03065-7 (PMC5809604; doi:10.1038/s41467-018-03065-7)
Supplement: Supplementary file 3 — Description of Additional Supplementary Files [file 41467_2018_3065_MOESM3_ESM.pdf]

Description of Supplementary Files

File Name: Supplementary Data 1
Description: **Sample size for all experiments.** The sample size for all experiments carried out in this work is shown in **Supplementary Data 1**, indicating the figure where the results are presented.
